# Applying infectious disease forecasting to public health: a path forward using influenza forecasting examples

**DOI:** 10.1186/s12889-019-7966-8

**Published:** 2019-12-10

**Authors:** Chelsea S. Lutz, Mimi P. Huynh, Monica Schroeder, Sophia Anyatonwu, F. Scott Dahlgren, Gregory Danyluk, Danielle Fernandez, Sharon K. Greene, Nodar Kipshidze, Leann Liu, Osaro Mgbere, Lisa A. McHugh, Jennifer F. Myers, Alan Siniscalchi, Amy D. Sullivan, Nicole West, Michael A. Johansson, Matthew Biggerstaff

**Affiliations:** 1 Influenza Division, National Center for Immunization and Respiratory Diseases, Centers for Disease Control and Prevention, Atlanta, GA 30329 USA; 2 Oak Ridge Institute for Science and Education, United States Department of Energy, Oak Ridge, TN 37830 USA; 3 Department of International Health, Johns Hopkins Bloomberg School of Public Health, Johns Hopkins University, Baltimore, MD 21205 USA; 4 Infectious Disease Program, Council of State and Territorial Epidemiologists, Atlanta, GA 30345 USA; 5 PHI/CDC Global Health Fellowship Program, Public Health Institute, Oakland, CA 94607 USA; 6 Florida Department of Health in Polk County, Bartow, FL 33830 USA; 7 Epidemiology, Disease Control, and Immunization Services, Florida Department of Health in Miami-Dade County, Miami, FL 33126 USA; 8 Bureau of Communicable Disease, New York City Department of Health and Mental Hygiene, Queens, New York, NY 11101 USA; 9 New York University, New York, NY 100003 USA; 10 Office of Science, Surveillance, and Technology, Harris County Public Health, Houston, TX 77027 USA; 11 Disease Prevention and Control Division, Houston Health Department, Houston, TX 77054 USA; 12 Communicable Disease Service, New Jersey Department of Health, Trenton, NJ 08608 USA; 13 Infectious Diseases Branch, California Department of Public Health, Richmond, CA 94804 USA; 14 Infectious Disease Section, Epidemiology & Emerging Infections Program, State of Connecticut Department of Health, Hartford, CT 06134 USA; 15 Division of Prevention and Community Health, Washington State Department of Health, Olympia, WA 98504 USA; 16 Acute and Communicable Disease Prevention, Oregon Health Authority, Portland, OR 97232 USA; 17 Division of Vector-Borne Diseases, National Center for Emerging and Zoonotic Infectious Diseases, Centers for Disease Control and Prevention, San Juan, PR 00920 USA

**Keywords:** Decision making, Disease outbreaks, Emergency preparedness, Forecast, Infectious disease, Influenza, Pandemic

## Abstract

**Background:**

Infectious disease forecasting aims to predict characteristics of both seasonal epidemics and future pandemics. Accurate and timely infectious disease forecasts could aid public health responses by informing key preparation and mitigation efforts.

**Main body:**

For forecasts to be fully integrated into public health decision-making, federal, state, and local officials must understand how forecasts were made, how to interpret forecasts, and how well the forecasts have performed in the past. Since the 2013–14 influenza season, the Influenza Division at the Centers for Disease Control and Prevention (CDC) has hosted collaborative challenges to forecast the timing, intensity, and short-term trajectory of influenza-like illness in the United States. Additional efforts to advance forecasting science have included influenza initiatives focused on state-level and hospitalization forecasts, as well as other infectious diseases. Using CDC influenza forecasting challenges as an example, this paper provides an overview of infectious disease forecasting; applications of forecasting to public health; and current work to develop best practices for forecast methodology, applications, and communication.

**Conclusions:**

These efforts, along with other infectious disease forecasting initiatives, can foster the continued advancement of forecasting science.

## Background

A forecast is a quantitative, probabilistic statement about an unobserved event, outcome, or trend and its surrounding uncertainty, conditional on previously observed data (N. Reich, personal communication). Perhaps the most familiar forecasts are for daily weather and severe storms, where accurate forecasts are used to protect life and property [1–3]. Similarly, decision makers could potentially use infectious disease forecasts to prepare for and prevent illness, hospitalization, and death, as well as the economic burden, experienced during infectious disease epidemics [4–6].

During a public health emergency response, leaders must make numerous critical decisions [4, 7]. These decisions are often reactive, occur in a rapidly changing environment where there is little or incomplete information available, and may be biased [8, 9]. Although traditional surveillance systems provide regular data updates, these systems are inherently retrospective and delayed, limiting their utility for real-time decision making and planning. Nowcasting – estimating present conditions or those immediately expected – and forecasting could help fill these gaps by providing guidance for the utility, scale, and timing of prevention strategies [4]. For example, during an influenza season, the coordination and communication of prevention strategies – such as vaccination and antiviral treatment – supports the deployment and management of important public health resources [7].

However, the use of infectious disease forecasts for decision making is challenging because most existing infectious diseases forecasts are not standardized, not validated, and can be difficult to communicate to non-scientific audiences. Forecasts may fail to address outcomes that are relevant for public health responders [10]. To address these limitations, the Centers for Disease Control and Prevention’s (CDC) Influenza Division (CDC/ID) and Division of Vector-Borne Diseases launched the Epidemic Prediction Initiative (EPI) in December 2014 [11, 12]. By bringing together public health officials and researchers from academia, industry, and government in an open forecasting project, EPI develops tools to address specific forecasting problems relevant to public health. EPI has hosted several challenges for predicting trends in influenza and other infectious diseases, addressing specific forecasting needs by engaging decision-makers and researchers in real-world forecasting scenarios (Table 1). These challenges provide participants experience in real-time forecasting, as well as experience in communicating results to public health practitioners. These challenges also offer a unique opportunity to evaluate forecast performance across different targets, seasons, geographic locations, and methods. Results from this evaluation enable researchers to prioritize future lines of inquiry and help decision-makers understand the strengths and limitations of current forecasts. These challenges are critical to developing a network of modelers capable of providing nowcasts and forecasts that public health officials can use.

**Table 1:** Summary of Completed and Planned EPI Forecasting Challenge Designs as of August 2019

Challenge Name	Health Outcome of Interest	Year(s)	Target(s)
Predict the Influenza Season Challenge	ILI in the United States at the national/regional level	2013–14	Season onset, peak week, peak intensity, season duration
FluSight 2014–15	ILI in the United States at the national/regional level	2014–15	Season onset, peak week, peak intensity, weekly ILI percent 1–4 weeks ahead
Dengue Forecasting Project	Dengue cases in Iquitos, Peru and San Juan, Puerto Rico	2015	Timing of peak incidence, maximum weekly incidence, total number of cases in a transmission season
FluSight 2015–16	ILI in the United States at the national/regional level	2015–16	Season onset, peak week, peak intensity, weekly ILI percent 1–4 weeks ahead
FluSight 2016–17	ILI in the United States at the national/regional level	2016–17	Season onset, peak week, peak intensity, weekly ILI percent 1–4 weeks ahead
FluSight 2017–18	ILI in the United States at the national/regional level	2017–18	Season onset, peak week, peak intensity, weekly ILI percent 1–4 weeks ahead
State FluSight 2017–18	ILI in the United States at the state/territory level	2017–18	Peak week, peak intensity, weekly ILI percent 1–4 weeks ahead
Influenza Hospitalizations 2017–18	Influenza hospitalizations in the United States	2017–18	Peak week, peek weekly hospitalization rate, weekly hospitalization rates 1–4 weeks ahead
FluSight 2018–19	ILI in the United States at the national/regional level	2018–19	Season onset, peak week, peak intensity, weekly ILI percent 1–4 weeks ahead
State FluSight 2018–19	ILI in the United States at the state/territory level	2018–19	Peak week, peak intensity, weekly ILI percent 1–4 weeks ahead
Influenza Hospitalizations 2018–19	Influenza hospitalizations in the United States	2018–19	Peak week, peek weekly hospitalization rate, weekly hospitalization rates 1–4 weeks ahead
*Aedes* Challenge 2019	*Aedes aegypti* or *Ae. Albopictus* (vectors of chikungunya, dengue, yellow fever, and Zika viruses)	2019	Monthly presence of *Aedes aegypti* or *Ae. albopictus*
FluSight 2019–20	ILI in the United States at the national/regional level	2019–20 (future)	Season onset, peak week, peak intensity, weekly ILI percent 1–4 weeks ahead
State FluSight 2019–20	ILI in the United States at the state/territory level	2019–20 (future)	Peak week, peak intensity, weekly ILI percent 1–4 weeks ahead
Influenza Hospitalizations 2019–20	Influenza hospitalizations in the United States	2019–20 (future)	Peak week, peek weekly hospitalization rate, weekly hospitalization rates 1–4 weeks ahead

The Council of State and Territorial Epidemiologists (CSTE) began collaborating with EPI in 2017 to achieve the following goals: improve the understanding of EPI forecasting activities among state and territorial public health officials, align EPI forecasts with the needs of those officials, and explore how forecasting can be more effectively integrated into public health decision-making. To this end, CDC and CSTE jointly host monthly workgroup meetings to discuss forecast accuracy and validation metrics, visualization and communication, collaboration and partner engagement, state and local health department perspectives, pilot projects, and other topics as they arise. Using seasonal influenza forecasting as an example, we review in this paper key considerations for infectious disease forecasts and lessons learned identified through this collaboration.

## Types of models and data sources used for forecasting

Mathematical models have long been used to study how humans, pathogens, and other hosts interact in infectious disease outbreaks to help identify ways to prevent or control them [13–16]. Many of these approaches have recently been adapted to generate forecasts of influenza outbreaks [17–21]. Table 2 presents the major modeling approaches that have been used to generate influenza outbreak forecasts.

**Table 2:** Major modeling approaches used to generate influenza outbreak forecasts*

Approach	Description	Strengths	Limitations
Agent-based models	These are computational systems in which persons are treated as individual agents that can interact with other agents and their environment based on specific rules.	These models have been used to address questions relating to the impact of control measures and changes in individual behavior during an outbreak. They allow for interactions between individuals and between individuals and their environments, and can therefore enable the forecasting of influenza dynamics under different intervention and resource allocation scenarios.	One difficulty in applying these models is the assumptions under which they operate, compounded by our limitations in understanding human behavior and contact networks. They are also computationally challenging and often require supercomputers.
Compartmental models	These models divide the population into compartments based on disease states and define rates at which individuals move between compartments. Examples include susceptible–infectious–recovered (SIR) and susceptible–exposed–infectious–recovered (SEIR) models.	Compartmental models are attractive due to their simplicity and well-studied behavior. These models are typically extended by defining multiple compartments to introduce subpopulations, or used in combination with other approaches, such as particle filtering, for influenza forecasting [20, 21].	The usual fully mixed, homogenous population assumption fails to capture the differences in contact patterns for different age groups and environments.
Ensemble models	Ensemble modeling is the process of running two or more models and synthesizing the results into a single forecast with the intent of improving the accuracy. The individual models may be nearly identical to each other or may differ greatly.	Ensemble models typically predict future observations better than a single model. Individual models in the ensemble can be weighted using recent or historical performance, or using a more complex algorithm.	The choice of which forecasts to include and how to weight the individual forecasts in the final ensemble may vary and is not standardized for infectious disease forecasting.
Metapopulation models	In between agent-based and compartmental models, populations are represented in structured and separated discrete patches and subpopulations interact through movement. Epidemic dynamics can be described within patches using clearly defined disease states such as in compartmental models.	The detailed mobility networks used in some of these models can enable reliable description of the diffusion pattern of an ongoing epidemic. These models have also been used to evaluate the effectiveness of various measures for controlling influenza epidemics.	Similar to agent-based models, empirical measurement or assumptions concerning interactions and movement is challenging.
Method of analogs	The method of analogs is a nonparametric forecasting approach. Forecasting is based on matching current influenza patterns to patterns of historical outbreaks.	The onset of seasonal influenza epidemics varies from year to year in most countries in the Northern hemisphere. As the method of analogs is nonparametric, it does not require explicit assumptions about underlying distributions or seasonality.	These forecasts rely on historical data which are often limited or not available. Limitations include the difficulty in finding similar patterns from historical outbreaks.
Time series models	These models typically use the Box-Jenkins approach and assume that future values can be predicted based on past observations.	Can capture lagged relationships that usually exist in periodically collected data. In addition, temporal dependence can also be represented in models that are capable of capturing trend and periodic changes.	Influenza activity is not consistent from season to season, which could impose limitations to these methods.

*Adapted from Nsoesie et al., 2014 [19]

**Fig. 1:**
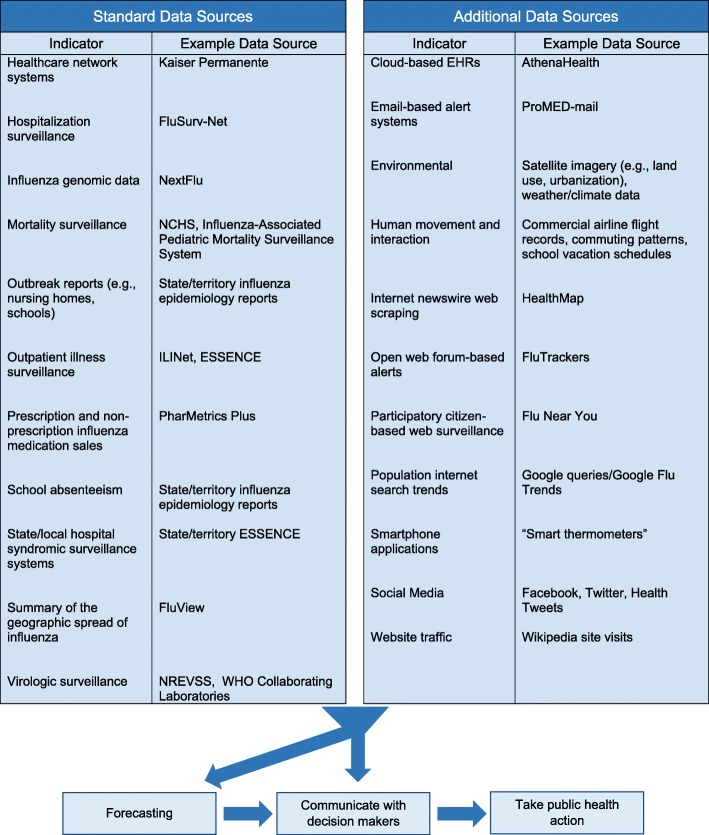
The use of trade names is for identification only and does not imply endorsement by the Centers for Disease Control and Prevention and/or the Council for State and Territorial Epidemiologists

While each approach has its own strengths and limitations, they are often tailored to specific forecasting targets based on the types of data that are available (Fig. 1).

## FLUSIGHT: influenza forecasting challenges

Human influenza – both seasonal and pandemic – is a major public health issue due to the continued emergence of novel genetic strains [22, 23]. Influenza causes substantial health and economic burden in the United States [24, 25], but the magnitude and timing of influenza seasons vary from year to year [26–29], making the annual impact difficult to predict at the beginning of each season. CDC/ID began working in 2013 to advance influenza disease forecasting efforts by engaging members of the scientific community who were already developing methods to predict influenza activity [30]. This collaboration launched with the “Predict the Influenza Season Challenge” (now referred to as EPI’s “FluSight”), a competition in which participants predicted the dynamics of the 2013–14 influenza season on a weekly basis as new data became available. Every season since, FluSight has hosted challenges to prospectively forecast the timing, intensity, and short-term trajectory (including nowcasts) of influenza-like illness (ILI) activity in the United States and the 10 Health and Human Service Regions [31] at weekly increments [32, 33]. The challenges run for one full influenza season, from late October/early November to mid-May of the next year. For example, the 2018–19 season challenge began October 29, 2018 and ended May 13, 2019. Starting in the 2017–18 influenza season, FluSight has also hosted pilots of forecasts of ILI at the state level and forecasts for confirmed influenza hospitalizations at the national level based on data from the Influenza Hospitalization Surveillance Network (FluSurv-NET)**.** Since the 2015–16 influenza season, EPI has posted the real-time influenza forecasts online [12]. The intent of FluSight is to better equip stakeholders to produce and use forecasts to guide public health decisions during influenza seasons and help inform forecasting in the event of an influenza pandemic.

## Forecast targets

Forecast targets are the outcomes being predicted. FluSight ILI national, regional, and state targets are currently based on data from the CDC’s U.S. Outpatient Influenza-like Illness Surveillance Network (ILINet), which includes data from the 1997–98 season to the present [34]. Currently, ILINet comprises more than 3500 enrolled outpatient healthcare providers around the country. Each week, approximately 2200 of these providers report data to CDC on the number of patients with ILI and the total number of patients seen in their practices [35]. While the representativeness and timeliness of ILINet data can vary by location across the United States and over time within the same location, ILINet has shown itself to be a useful indicator of influenza season timing and intensity, and is appropriate for national-level users and may be appropriate for state and local-level users. It also has the advantage of comprising a robust amount of historic data from which forecasters can draw upon.

**Fig. 2:**
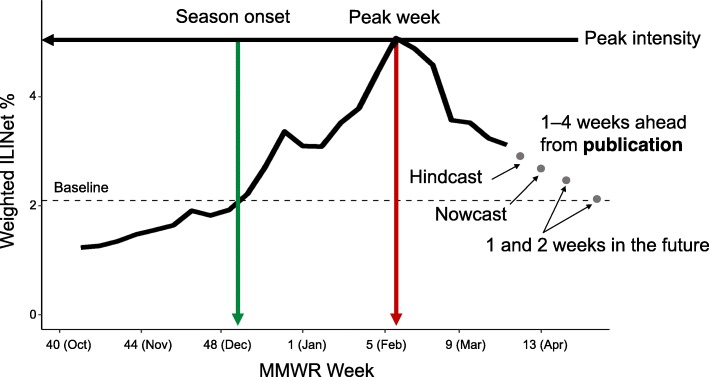
The Morbidity and Mortality Weekly Report (MMWR) week is the week of the epidemiologic year for which the National Notifiable Diseases Surveillance System (NNDSS) disease report is assigned by the reporting local or state health department for the purposes of disease incidence reporting and publishing [36]. Values range from 1 to 53, although most years consist of 52 weeks. The weeks shown in the figure above are for example only, as MMWR weeks and corresponding calendar date may shift year to year

Forecast targets should have specific quantitative definitions and be selected to address specific public health needs. For example, the current FluSight forecast targets include both seasonal and short-term targets, which are chosen to help public health officials understand the characteristics of the current influenza season relative to previous ones (Table 1, Fig. 2). The seasonal targets are onset, peak week, and peak intensity. For FluSight, these definitions rely on the ILINet percentage of visits for ILI, weighted by state population. Baseline ILI is determined by calculating the mean percentage of patient visits for ILI during non-influenza weeks for the previous three seasons and adding two standard deviations [35]. When the ILINet percentage exceeds baseline, influenza is likely circulating in the population [37]. Therefore, the season onset target is defined as the first week in the season when the weighted ILINet percentage is at or above baseline and remains above baseline for at least two additional weeks. Peak week is the week when the weighted ILINet percentage is the highest, and the peak intensity is the highest value that the weighted ILINet percentage reaches during the season. Short-term targets are forecasts of the weighted ILI percentage one, two, three, and four weeks in advance of its publication. Due to the delay in reporting (e.g., data for week 50 are published in week 51 and forecasts using those data are made in week 52), the 1-week ahead target forecasts the ILI percentage for the previous week (a hindcast); the 2-weeks ahead target forecasts the ILI percentage for the present week (a nowcast); and the 3-weeks and 4-weeks ahead target forecast the ILI percentage one week and two weeks in the future respectively.

## Forecast evaluation

Measuring the accuracy of infectious disease forecasts is critical for their applications in public health. Metrics for assessing accuracy often focus on error, which is the difference between a predicted outcome and the observed outcome [38]. Error-based metrics are attractive because they can be framed in the scale of the target (e.g., the predicted peak week was one week before the observed peak week). However, measuring accuracy in this way neglects a critical aspect of forecasts, which is the confidence (or probability) that the forecast places on being correct. Forecasts with confidence measures provide the public health decision-maker a more useful product [39]. For example, weather forecasts ascribe confidence when they provide a daily probability of rain.

FluSight evaluates forecasts as a set of probabilities of all the different possible outcomes. For example, timing of the peak of seasonal influenza could happen during any week of the year [36]. Forecasts, therefore, specify the probability of the peak occurring in each week (e.g., the probability of the peak occurring in Week 3 is 0.2, or a 20% chance), and forecasts follow the rules of a probability distribution. For example, a probability of 0.1 for Week 1, 0.7 for Week 2, and 0.2 for Week 3 indicates that there is a 100% chance of the peak between Weeks 1–3, and no chance of the peak occurring before Week 1 or after Week 3.

FluSight also evaluates forecast calibration. Calibration is an indicator of reliability in assigning probabilities and refers to the agreement between observed outcomes and predictions [40]. For example, when a forecast says there is a 0.2 probability (i.e., 20% chance) of rain, it should rain approximately 20% of the days when similar atmospheric conditions occur. To evaluate calibration, FluSight groups forecasts by probabilities (e.g., those with a probability of 0.1 to 0.2 or 10–20%) and assesses how often those forecasts were correct. Although both high and low probability forecasts can be useful (e.g., there is a 10% chance that the peak will occur next week), all forecasts need to be well calibrated.

When determining the best forecasting model, FluSight limits its evaluations to those forecasts produced during critical periods of the influenza season. For example, the evaluation period for season onset is the first week of the challenge through six weeks after the observed onset week. This evaluation period varies by forecasting target and geographic region, representing the weeks when the forecasts are most useful. FluSight compares forecasts by taking the logarithm of the probability assigned to values within a window containing the eventually observed outcome. This value is called the logarithmic score and, when averaged across various forecasts (e.g., weeks, targets, and geographic regions), indicates which set of forecasts provided the highest confidence in the true outcome [41]. FluSight converts the logarithmic score to a “forecast skill” metric by exponentiating the average logarithmic score. Forecast skill indicates the average probability assigned to the observed outcome and is on a scale of 0 to 1. For example, a skill of 0.7 indicates a set of forecasts, on average, assigned a 0.7 probability of occurrence to the probability bin containing the observed outcome during the evaluation period. Forecast skill is the key metric for evaluating overall forecast accuracy and is calculated after the evaluation period has concluded and the true target value has been observed. As the FluSight challenge evolved, organizers at CDC implemented this “moving window” score to achieve a balance between “strictly proper scoring and high resolution binning (e.g. at 0.1% increments for ILI values) versus the need for coarser categorizations for communication and decision-making purposes” [42].

FluSight does not currently use any threshold for forecast skill when considering whether a forecast is useful because forecast skill depends on the forecast target, geographic scale, and the decision context. Instead, FluSight forecasts are compared to each other, as well as to a historic average forecast. The historic average forecast only uses ILINet data from previous seasons, providing a surveillance benchmark to place forecast accuracy into context. Quantifying the accuracy of forecasts and comparing them is critical for acceptance, as historical performance provides an evidence base for decision-makers who may use those forecasts in the future. Accuracy can vary systematically by season, region, and target type. Therefore, data should be available from multiple seasons with different characteristics (e.g., early vs. late onset, high vs. low severity, one type/subtype vs. another), multiple geographic areas, and be stratified by target. Importantly, forecast accuracy may be lower in atypical seasons when historical data are less relevant, for example, during high severity seasons or seasons with a late peak.

## Results from FLUSIGHT challenges: 2013–14 through 2017–18 seasons

The majority of participants in the FluSight challenges used a combination of historical influenza data, Twitter, Google Flu Trends, and weather data sources to inform their models; approximately half of the forecasts employed statistical methods and half employed mechanistic models (e.g., compartmental models) [30, 32, 33, 43, 44]. Table 3 summarizes the results of the 2013–14 [30], 2014–15 [32], 2015–16 [33], 2016–17 [43, 44], and 2017–18 [43, 44] challenges; results from the 2016–17 and 2017–18 challenges have not been published, and results from 2018 to 19 are still being evaluated as of August 2019. Overall, seasonal forecasts tended to see improvements in forecast skill as the season progressed closer to when the true targets were observed. Short-term prediction skills and the accuracy of point forecasts were highest for one-week ahead forecasts and declined for the two-, three-, and four-week ahead forecasts. Short-term skills also declined around the period of peak influenza activity. During the 2013–14 challenge, forecast evaluation was qualitative. In 2014–15, FluSight introduced the logarithmic scoring rule to quantify forecast skill; it was modified and finalized prior to the start of the 2015–16 challenge and has been implemented every season since. The same team had the highest overall forecast skill for the 2014–15 through 2017–18 seasons (Table 3) [45]. Moreover, ensemble models, either submitted by teams or created as an average of all submitted forecasts, consistently outperformed both individual model forecasts and forecasts based on historical patterns alone.

**Table 3:** Summary of results from the FluSight influenza forecast challenges*

	2013–14 season	2014–15 season	2015–16 season	2016–17 season	2017–18 season
Number of participating teams	9	5	11	21	22
Number of submitted forecasts^†^	13	7	14	28	29
Season onset top skill	N/A**	0.41	0.18	0.78	0.69
Peak week top skill	N/A	0.49	0.20	0.49	0.50
Peak intensity top skill	N/A	0.17	0.66	0.36	0.26
1-week ahead top skill	N/A	0.43	0.89	0.60	0.54
2-weeks ahead top skill	N/A	0.36	0.76	0.46	0.37
3-weeks ahead top skill	N/A	0.37	0.66	0.41	0.29
4-weeks ahead top skill	N/A	0.35	0.58	0.38	0.26
Overall top performing team	Columbia University	Delphi group, Carnegie Mellon University	Delphi group, Carnegie Mellon University	Delphi group, Carnegie Mellon University	Delphi group, Carnegie Mellon University

*Skill scores for 2016–17 and 2017–18 challenges have not been published. Results from 2018 to 19 challenge are not complete as of August 2019

†Number of submitted forecasts do not include the unweighted average ensemble or historical average forecasts

**The logarithmic scoring rule used to determine forecast skill scores was not introduced until the second year of the challenge (2014–15). Skill scores for the challenge pilot (2013–14) are therefore not available

## Applications of forecasting for public health decision-making

Preparation for and response to disease outbreaks and epidemics are essential public health functions; yet decision-makers often do not have a standardized and validated way to assess when and where increases in disease will occur, how long they will last, or when they will resolve. From disease control to recovery activities, actions taken during a response rely on decisions made along a spectrum of short- to long-term planning horizons. Forecasting could support this spectrum, and the annual FluSight challenges demonstrate great potential for applying these forecasts in real-world settings [12]. For example, forecasts are currently used to inform CDC’s routine influenza season risk communication talking points provided to partners, weekly summaries presented to CDC leadership, and public messaging regarding the timing of the influenza season and how the public can protect themselves and their families [45, 46]. In addition, weekly forecasts are distributed to state and local public health officials in real-time during the challenges through CSTE/CDC Forecasting Workgroup emails and monthly conference calls. During these calls, CDC, CSTE, state and local public health officials, and forecasters discuss forecast results, utility, and methods to improve forecast visualization and communication.

The potential uses of infectious disease forecasts extend beyond communication, both in seasonal and emergency situations. Forecasts could provide information useful for risk management, such as informing messages to healthcare providers (including hospitals) regarding appropriate treatment for patients (e.g. antiviral treatment in the case of influenza). Forecasts could also aid in preparation for surge capacity and hospital resource management by anticipating staffing needs and resource usage, potentially guiding the allocation and deployment of human resources and treatment inventory. Finally, forecasts could guide community mitigation strategies, such as school closures during pandemics. While public health emergencies and pandemics may be fundamentally different from annual influenza seasons and seasonal forecast accuracy may not be a predictor of pandemic forecast accuracy, the FluSight challenges have helped develop a network of modelers more capable of providing nowcasts and forecasts that public health officials can use during a future pandemic.

Although quantitative data on forecast use is limited to the abovementioned examples, CDC and CSTE are collaborating on additional ongoing projects to identify, evaluate, and quantify how the FluSight forecast results are being utilized by stakeholders (e.g., state influenza coordinators).

## Communication strategies

**Fig. 3:**
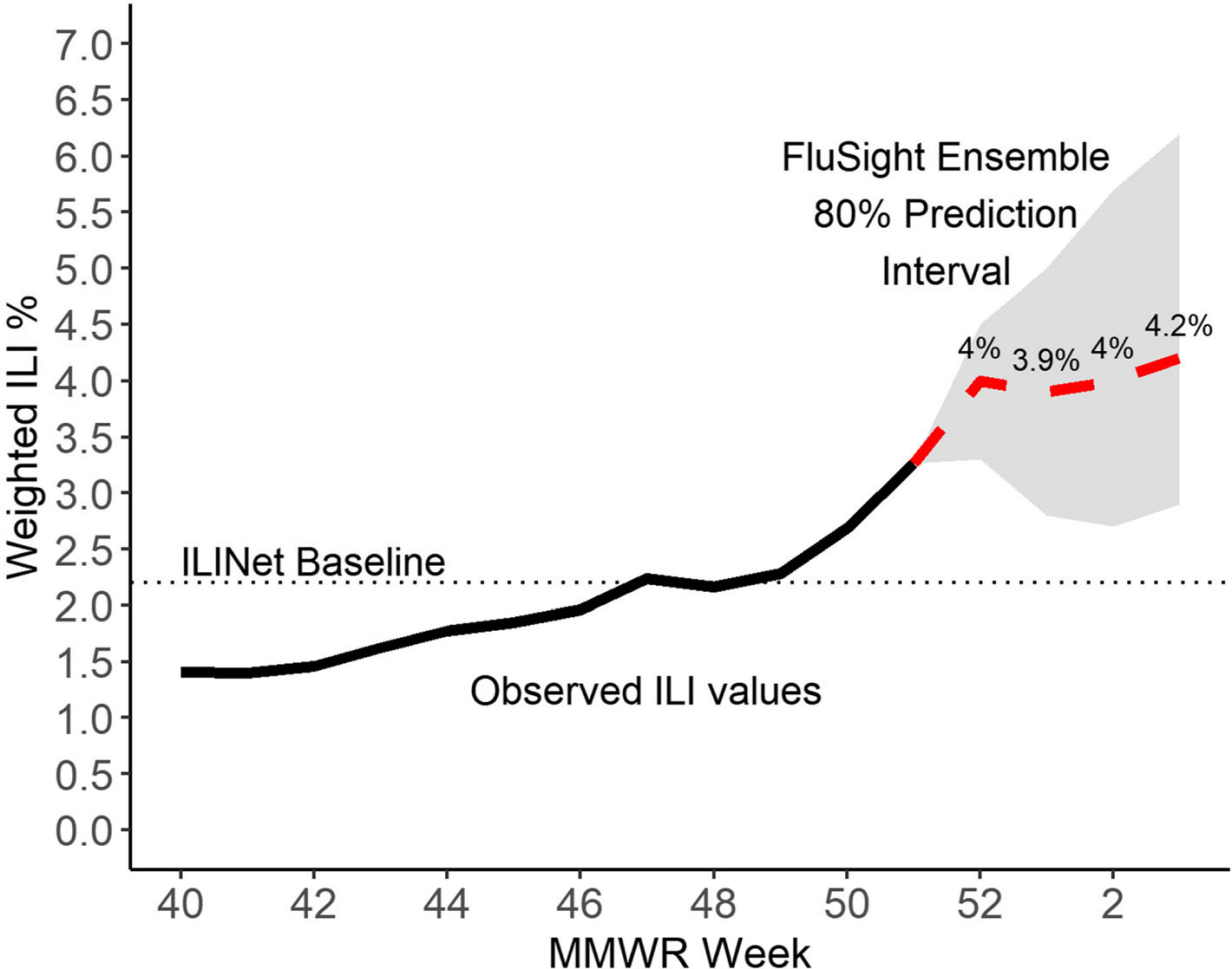
Predictions for national ILI percentage published for Week 52 through Week 3 (1-, 2-, 3-, and 4-weeks ahead, respectively) and associated 80% prediction interval

Forecasts could be a valuable resource for infectious disease outbreak preparation and response. However, this vision not only requires accurate forecasts but also effective communication tools such that key stakeholders – e.g., public health officials, healthcare providers, the media, and the public – can interpret, understand, and act quickly and appropriately. Therefore, the utility of a forecast (even a perfectly accurate one) is directly tied to how successful the forecasters and epidemiologists are at communicating methodology and interpretations, including forecast confidence and uncertainty. One method for communicating information to end users that has increased in popularity is data visualization tools [47]. An example of one of the current methods of presenting outputs from the 2018–19 FluSight Challenge is presented in Fig. 3. Additionally, consistent dialogue, preferably occurring outside of emergency conditions, should address how to appropriately interpret forecasting information, as well as the strengths and limitations of forecasting in general. Dialogue is essential to keep decision-makers informed and to ensure that forecast products are designed to support public health activities.

Multiple efforts have been undertaken to improve forecast communication with stakeholders. A recent analysis by IQT Labs, in collaboration with CDC/ID, found that in communicating forecast results, there is a need to strike the balance between the number of forecasts presented and representing forecast uncertainty and confidence in a way that is both accurate and clear to the user [47]. This work has the potential to help identify best practices for visualizing forecast data and provide a framework for approaching future communications and visualization efforts. However, work is still ongoing in this area and clear recommendations are not yet available. To this end, CDC has established two ongoing research projects. The first is a CSTE/CDC forecasting data visualization project to identify best communication practices, needs of stakeholders for forecast messaging, and useful communication products. The second is the development of a network of Forecasting Centers of Excellence to improve the accuracy and communication of real-time probabilistic forecasts for seasonal and pandemic influenza [48].

## Forecasting beyond influenza

In addition to influenza forecasting, EPI has also hosted forecasting challenges for dengue (Table 1). In tropical areas, the incidence of dengue, a mosquito-borne viral disease, follows seasonal transmission patterns similar to influenza, but every few years, there are much larger epidemics that overwhelm health systems [49, 50]. In 2015, several U.S. government agencies and the White House Office of Science and Technology Policy undertook a retrospective forecasting (i.e., forecasting past events using data only from time periods prior to those events) project to galvanize efforts to predict epidemics of dengue using the same basic framework that has been employed for influenza [51]. Forecasting targets included peak incidence, maximum weekly incidence, and the total number of cases in a transmission season. Researchers evaluated probabilistic forecasts using the logarithmic score. More information about this work is available on EPI’s website under “Dengue Forecasting Project.”

In March 2019, EPI launched the “*Aedes* Forecasting Challenge” to predict the spatiotemporal distribution of *Aedes aegypti* and *Ae. albopictus* mosquitoes in the United States (Table 1) [52]. This open forecasting challenge aims to predict the monthly presence of these species in a subset of U.S. counties during the 2019 calendar year, and uses data from 95 counties in eight states. Other opportunities to use forecasting to support public health decision-making in the U.S. include preparing for potential introduction and local transmission of chikungunya [53] or Zika virus [54]. Forecasts indicating likely increases in risk provide evidence to public health officials and other stakeholders to alert clinicians, communicate with the public, and plan mosquito surveillance and control activities.

Forecasting beyond influenza may focus on different targets and will require the inclusion of different datasets. However, such forecasts can be easily adapted to the EPI platform, as evidenced by the Dengue Forecasting Project and *Aedes* Forecasting Challenge. Lessons learned through the FluSight, dengue, and *Aedes* challenges, such as accuracy assessment, communication strategies, visualization, and public health implications will likely be broadly applicable to other infectious disease forecasts.

## Limitations

Despite advantages and potential applications, there are still a number of challenges and limitations to infectious disease forecasting. From a methodological perspective, each forecasting model will have its own inherent limitations specific to the method being used (Table 2). Furthermore, the influenza forecasting efforts described here mainly relied on data reported through ILINet, which captures reported cases of ILI and not laboratory-confirmed influenza. The influenza hospitalization forecast pilot launched during the 2017–18 season aims to address this limitation by forecasting an influenza-confirmed outcome (i.e., hospitalization). Hospitalization forecasts may prove to be a more robust forecasting target, as FluSight has access to high quality and reliable data regarding how hospitalized patients are identified and captured in FluSurv-NET. In addition, even though the United States has several established systems for conducting influenza surveillance, data availability and comparability limitations remain at the state and sub-state scale [55–57], potentially limiting the development and utility of forecasts for these jurisdictions. Similarly, reproducing the proposed methods of forecasting for other pathogens or countries may prove challenging if no historic dataset exists [13]. Furthermore, despite ongoing efforts to address knowledge gaps, at present, quantifiable data regarding how end users utilize forecast results are not available. Finally, as forecasting science grows and evolves, discussions regarding forecast ownership, funding, and comparability of methodological approaches will be needed.

## Technical support

In an effort to standardize language used in forecasting, we developed a glossary of commonly used terms (Table 4). Furthermore, stakeholders who wish to engage in the CSTE/CDC Forecasting Workgroup or who have specific requests for technical assistance should contact the CSTE/CDC Forecasting Workgroup (forecasting@cste.org).

**Table 4:** Glossary of terms commonly used in forecasting

Forecasting term	Forecasting term definition
Ensemble model	A model that incorporates two or more models into a single model.
Epidemic Prediction Initiative	A CDC initiative launched in 2014 that aims at improving the science and usability of epidemic forecasts by facilitating open forecasting projects with specific public health objectives.
FluSight Challenge	A multi-participant competition that began during the 2013–14 influenza season (then called the “Predict the Influenza Season Challenge”) to forecast the timing, intensity, and short-term trajectory of the influenza season.
Forecast	A quantitative, probabilistic statement about an unobserved event, outcome, or trend and its surrounding uncertainty, conditional on previously observed data.
Forecast accuracy	A measurement of how well the forecast matched the outcome once it has been observed. There are a number of ways forecast accuracy can be measured, but CDC uses the logarithmic score. For more information regarding logarithmic score, please see the definition below.
Forecast calibration	An indicator of reliability in assigning probabilities. For FluSight forecasts, calibration is evaluated by assessing how often forecasts were correct.
Forecast confidence	A characterization of the uncertainty in a forecast. The Epidemic Prediction Initiative requires that forecast confidence be expressed as a probability (e.g., a 0.2 probability or 20% chance that the peak week of the influenza season will be on week 2).
Hindcast	Forecast of past conditions, also known as “pastcast.” For example, due to delays in reporting and data accrual, the FluSight forecast for ILI outpatient visits “one week ahead” is actually a forecast for the previous calendar week.
ILI	Influenza-like illness, fever and either a cough or sore throat.
ILINet	US Outpatient Influenza-like Illness Surveillance Network; a surveillance system that accrues weekly data on the number of patients with ILI and the total number of patients seen in healthcare settings, reported by outpatient healthcare providers in the United States.
Logarithmic score	The logarithm of the probability assigned to the observed outcome averaged across various forecasts (e.g., weeks, targets, and geographic regions). Used to measure the accuracy of a forecast.
Nowcast	Forecast of current conditions. For example, due to delays in reporting and data accrual, the FluSight forecast for ILI outpatient visits “two weeks ahead” is actually a forecast for the current calendar week.
Onset	The start of sustained disease activity. As a seasonal target for FluSight forecasts, it is defined as the first week when the percentage of visits for ILI reported through ILINet reaches or exceeds the baseline value for three consecutive weeks. No onset is a possible outcome.
Peak intensity	The maximum weekly or monthly value that disease activity reaches. As a seasonal target for FluSight forecasts, it is defined as the highest numeric value that the weighted ILINet percentage reaches during a season.
Peak week	The week that disease activity reaches it maximum. As a seasonal target for FluSight forecasts, it is defined as the week during the influenza season when the weighted ILINet percentage is the highest. More than one peak week is a possible outcome.
Reliability	A measure of how well the forecasted probability of an event occurring matches the observed outcome. Reliability answers the question whether a forecast that assigns a probability of 0.2 observes the forecasted event 20% of the time. This is also known as forecast calibration.
Retrospective forecast	A forecast of a past event (e.g., past influenza or dengue seasons) using data only from time periods prior to the event.
Skill	The average confidence (or probability) that was assigned to the observed outcome.
Seasonal target	Forecasts for the overall influenza season characteristics. These forecasts currently include the onset week, peak week, and peak intensity.
Short-term target	Forecasts for the near-term trajectory of the influenza season. These forecasts currently include forecasts for influenza activity one, two, three, and four weeks ahead from the date of data publication.
Target	The outcome that a forecast is predicting.

## Conclusions

Accurate and timely infectious disease forecasts could inform public health responses to both seasonal epidemics and future pandemics by providing guidance for the utility, scale, and timing of prevention and mitigation strategies. Since the 2013–14 influenza season, FluSight has hosted collaborative challenges to forecast the timing, intensity, and short-term trajectory of ILI activity in the United States using data published in ILINet. These efforts, along with other infectious disease forecasting initiatives, can foster the continued advancement of forecasting science. Challenges and limitations exist with infectious disease forecasting, but these can be addressed through further research and the refinement of existing tools. To this end, EPI, CSTE, and other partners continue to work towards the development of best practices for forecast applications, methodology, and output communication. Despite current limitations, forecasting is a powerful tool to aid public health decision making.

## Data Availability

Not applicable.

## Abbreviations

CDC: Centers for Disease Control and Prevention
CDC/ID: Centers for Disease Control and Prevention, Influenza Division
CSTE: Council for State and Territorial Epidemiologists
EPI: Epidemic Prediction Initiative
FluSurv-NET: Influenza Hospitalization Surveillance Network
ILI: Influenza-like illness
ILINet: U.S. Outpatient Influenza-like Illness Surveillance Network
